# Mirna Expression in Glaucomatous and TGFβ2 Treated Lamina Cribrosa Cells

**DOI:** 10.3390/ijms22126178

**Published:** 2021-06-08

**Authors:** Navita N. Lopez, Rajiv Rangan, Abbot F. Clark, Tara Tovar-Vidales

**Affiliations:** Department of Pharmacology and Neuroscience, The North Texas Eye Research Institute, University of North Texas Health Science Center at Fort Worth, Fort Worth, 3500 Camp Bowie Blvd., Fort Worth, TX 76107, USA; navitalopez@my.unthsc.edu (N.N.L.); rajivrangan@my.unthsc.edu (R.R.); abe.clark@unthsc.edu (A.F.C.)

**Keywords:** optic nerve head, ONH, LC cells, miRNAs, miR-29, ECM

## Abstract

Glaucoma is a group of optic neuropathies that leads to irreversible vision loss. The optic nerve head (ONH) is the site of initial optic nerve damage in glaucoma. ONH-derived lamina cribrosa (LC) cells synthesize extracellular matrix (ECM) proteins; however, these cells are adversely affected in glaucoma and cause detrimental changes to the ONH. LC cells respond to mechanical strain by increasing the profibrotic cytokine transforming growth factor-beta 2 (TGFβ2) and ECM proteins. Moreover, microRNAs (miRNAs or miR) regulate ECM gene expression in different fibrotic diseases, including glaucoma. A delicate homeostatic balance between profibrotic and anti-fibrotic miRNAs may contribute to the remodeling of ONH. This study aimed to determine whether modulation of miRNAs alters the expression of ECM in human LC cells. Primary human normal and glaucoma LC cells were grown to confluency and treated with or without TGFβ2 for 24 h. Differences in expression of miRNAs were analyzed using miRNA qPCR arrays. miRNA PCR arrays showed that the miR-29 family was significantly decreased in glaucomatous LC cell strains compared to age-matched controls. TGFβ2 treatment downregulated the expression of multiple miRNAs, including miR-29c-3p, compared to controls in LC cells. LC cells transfected with miR-29c-3p mimics or inhibitors modulated collagen expression.

## 1. Introduction

The glaucomas are a heterogeneous group of optic neuropathies defined by an irreversible loss of vision. The cellular and molecular pathophysiology of glaucoma is complex, as multiple factors contribute to the etiology; however, a universal characteristic of all glaucomas is damage to the optic nerve head (ONH) and degeneration of retinal ganglion cell (RGC) axons.

Primary open-angle glaucoma (POAG) is the most prevalent subtype of glaucoma. Although it is a multifactorial disease, elevated intraocular pressure (IOP) is a strong risk factor for POAG development [1,2,3]. Electron micrographs from glaucoma donor eyes reveal the earliest detectable damage is at the lamina cribrosa (LC) of the ONH [4]. The LC is a distinct region of the ONH formed by successive, connective tissue plates that provide essential scaffolding and pores for unmyelinated RGC axons. The connective tissue plates are composed of extracellular matrix (ECM) macromolecules including collagen, elastin, proteoglycans and glycoproteins, which together provide the strength and elasticity of the LC [5,6,7]. The glaucomatous LC has notably increased collagen type IV deposition as well as disorganization of collagen type I fibrils and fragmentation of elastin [8,9,10,11,12,13,14]. A weakened LC can lead to excessive forces acting on RCC axons resulting in degeneration and RGC apoptosis.

Besides RGC axons, there are two major cell types in the ONH—ONH astrocytes and LC cells [15,16]. LC cells are mechanosensitive cells that interact with the surrounding ECM [17] and respond to mechanical strain by upregulating gene expression of growth factors, including transforming growth factor-beta 2 (TGFβ2) and ECM proteins such as collagen type IV [15]. Our research group and others have shown that expression of TGFβ2 is higher in glaucomatous LC tissue compared to normal age-matched controls [18,19] and is implicated in altered gene expression and increased ECM deposition in glaucoma [12,15,18,20]. Cultured LC cells secrete TGFβ2 and respond to exogenous TGFβ2 by activating the canonical Smad signaling pathway that increases the synthesis and secretion of ECM proteins [18], suggesting that these cells have an active role in the pathological remodeling of the glaucomatous LC. Mechanical stretch of cultured LC cells increases TGFβ2 expression [15].

We aimed to further explore the mechanisms involved in regulating ECM gene and protein expression in LC cells. microRNAs (miRNAs or miR) are non-coding regulatory RNAs that mediate post-transcriptional regulation of protein-coding genes, including the ECM. miRNAs are tightly regulated to maintain homeostasis; however, their expression is altered in fibrotic diseases, including glaucoma [21,22]. Furthermore, the expression of miRNAs is sensitive to growth factor signaling, including TGFβ [23]. TGFβ activation of SMADs, cofactors, and transcription factors can lead to transcriptional activation or inhibition of miRNA genes. For example, TGFβ signaling induces differentiation of myoblasts to myofibroblasts via SMAD3 binding to the miR-29 promoter and subsequent downregulation of miR-29 expression [24]. miRNAs can also be modulated at the post-transcriptional level by R-SMAD proteins, which recruit and interact with members of the miRNA processing complex, DROSHA and p68, and enhance cleavage of pri-miRNAs to mature miRNAs [25]. Therefore, miRNAs are important for achieving homeostatic regulation of TGFβ signaling.

Since TGFβ signaling can directly influence the expression of miRNAs and miRNAs regulate the translation of proteins, we hypothesized that TGFβ2 deregulates miRNA expression in LC cells and contributes to LC ECM remodeling. In this study, we used miRNA PCR arrays to determine miRNA expression in glaucomatous and TGFβ2 treated LC cells compared to control LC cells. We also evaluated the effects of miR-29 on collagen type I and IV expression in LC cells.

## 2. Results

### 2.1. Differentially Expressed miRNAs in POAG and TGFβ2 Treated LC Cells Compared to Non-Glaucomatous Control LC Cells

**Figure 1:**
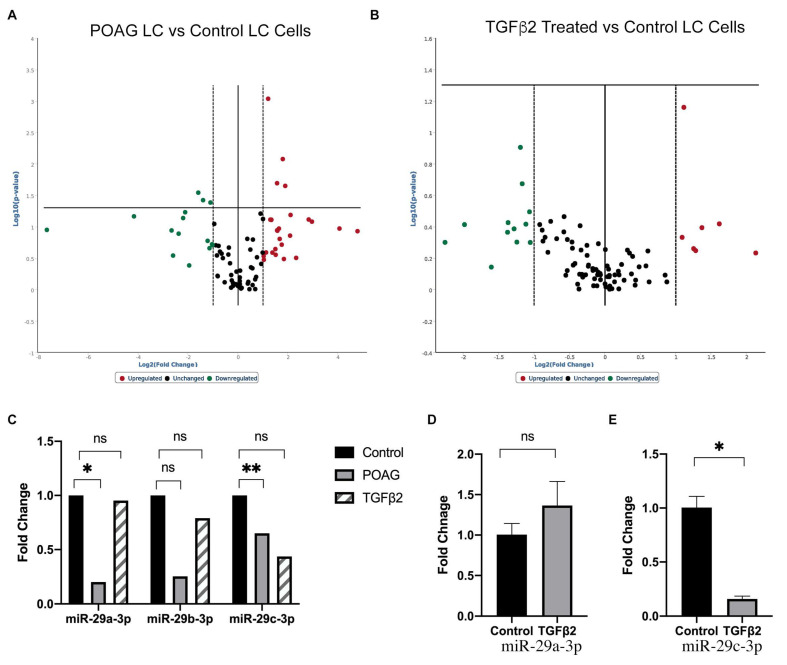
Analysis of differentially expressed mature miRNA in LC cells. Volcano plots of fibrosis pathway-related miRNAs in (**A**) normal and glaucoma primary human LC cells, or (**B**) normal human LC cells treated with or without TGFβ2 for 24 h. Upregulated miRNAs are shown in red to the right, and downregulated miRNAs are shown in green to the left. The horizontal bar indicates the threshold significance of *p* < 0.05. miRNAs were considered significantly upregulated/downregulated if they passed the threshold significance of *p* < 0.05 (horizontal line). (**C**) Expression of the miR-29 family in glaucomatous and TGFβ2 treated LC cells compared to controls. Evaluation of (**D**) miR-29a-3p or (**E**) miR-29c-3p expression in TGFβ2 treated LC cells analyzed by qPCR (*p* < 0.05), *n* = 3 biological replicates. * *p* < 0.05; ** *p* < 0.005.

We isolated and characterized primary LC cells from POAG and non-glaucomatous eyes. The expression of 88 miRNAs were analyzed in non-glaucomatous and POAG LC cell strains (Figure 1A; Supplementary Table S1). A volcano plot identified differentially expressed profibrotic or anti-fibrotic miRNAs with statistically significant differences in expression (Figure 1). Of these, seven were significantly differentially expressed compared to age-matched non-glaucomatous cells. Hsa-miR-150-5p, hsa-miR-338-5p, hsa-miR-382-5p, and hsa-miR-451a were significantly upregulated in POAG LC cells compared to non-glaucomatous LC cells (*n* = 3; *p* < 0.05). Hsa-miR-26b-5p, hsa-miR-29a-3p, and hsa-miR-29c-3p were downregulated in POAG LC cells compared to non-glaucomatous LC cells (*n* = 3; *p* < 0.05).

Mature miRNA expression was analyzed across three non-glaucomatous LC cell strains in response to TGFβ2 treatment (Figure 1B; Supplementary Table S2). miRNAs upregulated included miR-146b-5p, miR-20a-5p, miR-217, miR-324-5p, miR-328-3p, and miR-377-3p. miRNAs downregulated included miR-10a-5p, miR-122-5p, miR-146a-5p, miR-19b-3p, miR-200a-3p, miR29b/c-3p, and miR-449a. However, the miRNA expression changes in LC cells in response to TGFβ2 were not statistically significant.

Since the expression of miR-29c appeared to decrease in glaucomatous and TGFβ2 treated LC cells (Figure 1C), we further explored the role of this candidate miRNA in LC cells. A q-PCR analysis of LC cells treated with TGFβ2 showed no significant change in the expression of miR-29a-3p (Figure 1D); however, there was a significant decrease in the expression of miR-29c-3p (Figure 1E).

### 2.2. Effects of miR-29c-3p on the ECM

**Figure 2:**
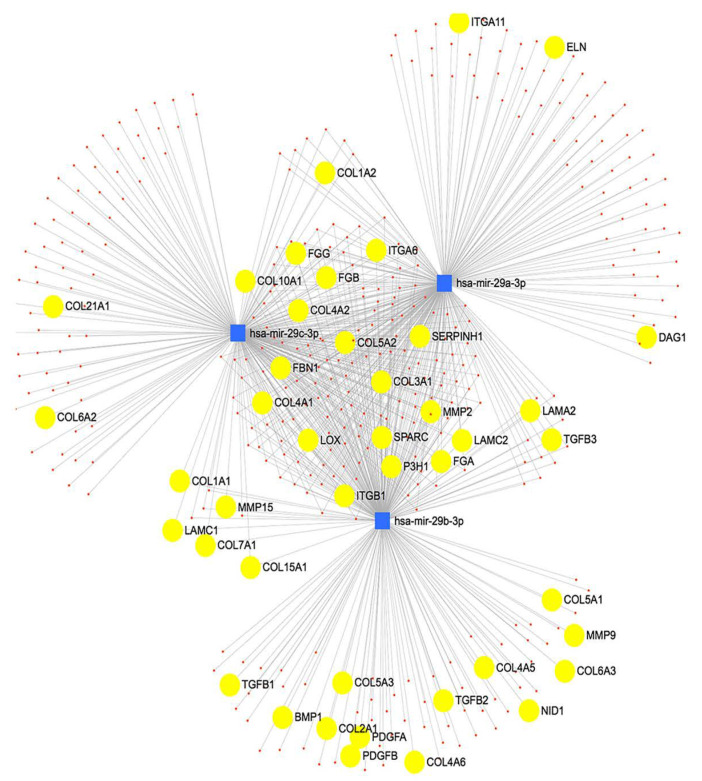
Network analysis of miR-29 regulated genes. miRNet was used to identify target genes regulated by the miR-29 family: miR-29a, miR-29b, and miR-29c. The miR-29 family interacts with several genes connected to ECM synthesis. miRNAs are represented in blue and target mRNAs are represented in yellow.

**Figure 3:**
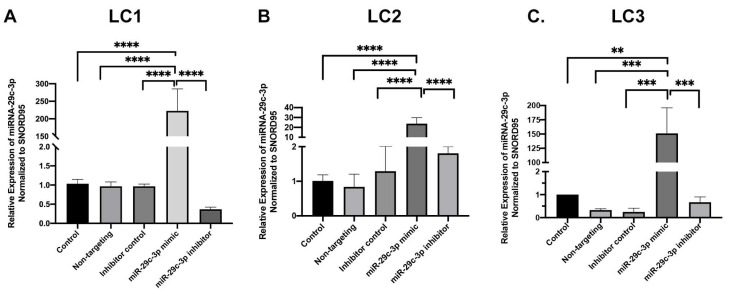
miRNA-29c-3p transfection efficiency in LC cells. Primary human LC cells were transfected with miR-29c-3p mimic (10 nm), miR-29c-3p inhibitor (10 nm), or non-targeting controls and transfection efficiency as determined by qPCR. SNORD95 was used as a normalizing control. (**A**–**C**) miRNA-29c-3p expression in each LC cell strain (LC1—donor 56 years old, LC2—donor 70 years old, and LC3—donor 74 years old). Error bars show the standard deviation (SD). One-way ANOVA analysis, Tukey’s multiple comparison test, *n* = 3 biological replicates, **** *p* < 0.0001; *** *p* < 0.0005; ** *p* < 0.005.

**Figure 4:**
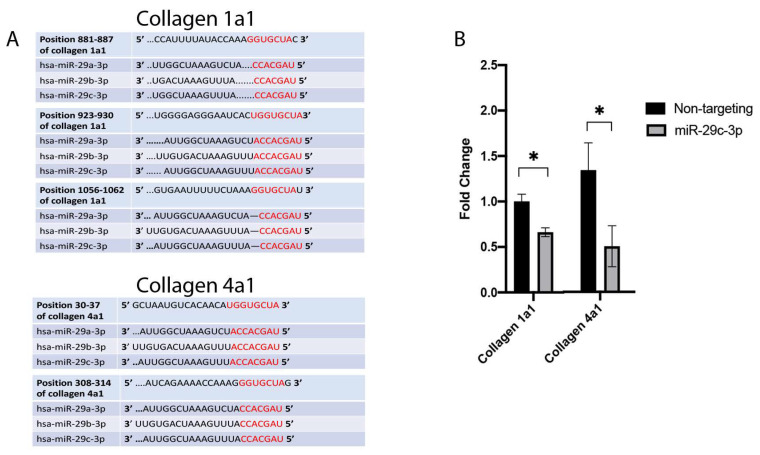
Collagens as prominent targets of the miR-29 family. (**A**) The TargetScan database was used to identify the 3′-UTRs of collagen 1a1 and collagen 4a1 as predicted binding sites for miR-29 family members (red). (**B**) Primary human LC cells were transfected with miR-29c-3p mimic (10 nm) or non-targeting control (10 nm). Expression of collagens type 1a1 and 4a1 were analyzed by qPCR. GAPDH was used as an internal control for normalization (* *p* < 0.05).

Using miRNet, we determined predicted genes and pathways associated with the miR-29 family. The miR-29 family targets genes that regulate the synthesis, organization, and degradation of ECM proteins, including *collagens* (Figure 2). We transfected the candidate miR-29c-3p mimic or inhibitor to confirm efficient upregulation and downregulation, respectively, of miR-29c-3p expression in LC cell strains (Figure 3 A–C). We next sought to identify the predicted miRNA-29 binding sites in the 3′-UTRs of collagen 1a1 and collagen 4a1 (Figure 4A) using the TargetScan database. We also sought to validate collagen 1a1 and collagen 4a1 with overexpression of miR-29c-3p. Overexpression of miR-29c-3p resulted in a decrease in collagen 1a1 and collagen 4a1 gene expression compared to the non-targeting control (Figure 4B).

### 2.3. Effects of miR-29c on TGFβ2 Induced-ECM Proteins

**Figure 5:**
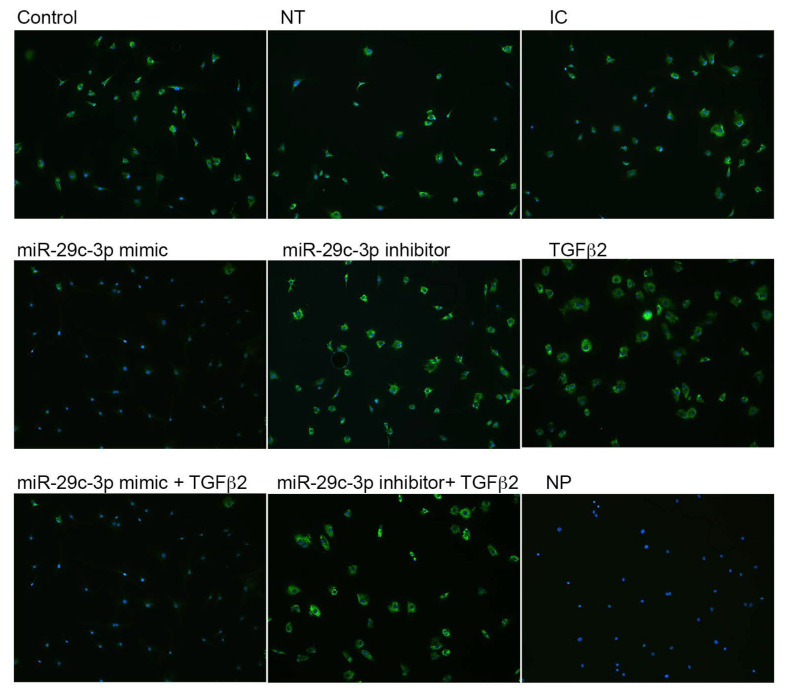
TGFβ2 and miR-29c-3p regulate collagen I expression in LC cells. Primary human LC cells were transfected with miR-29c-3p mimic (10 nM), inhibitor (10 nM), inhibitor control (IC; 10 nM), or non-targeting (NT; 10 nM) control in the presence or absence or TGFβ2 (5 ng/mL) for 72 h. Cells were fixed and immunolabelled with anti-collagen type I antibody (green). Nuclei (blue) were stained with DAPI. NP = no primary Ab control. Representative images are shown. Images taken at 100× magnification.

**Figure 6:**
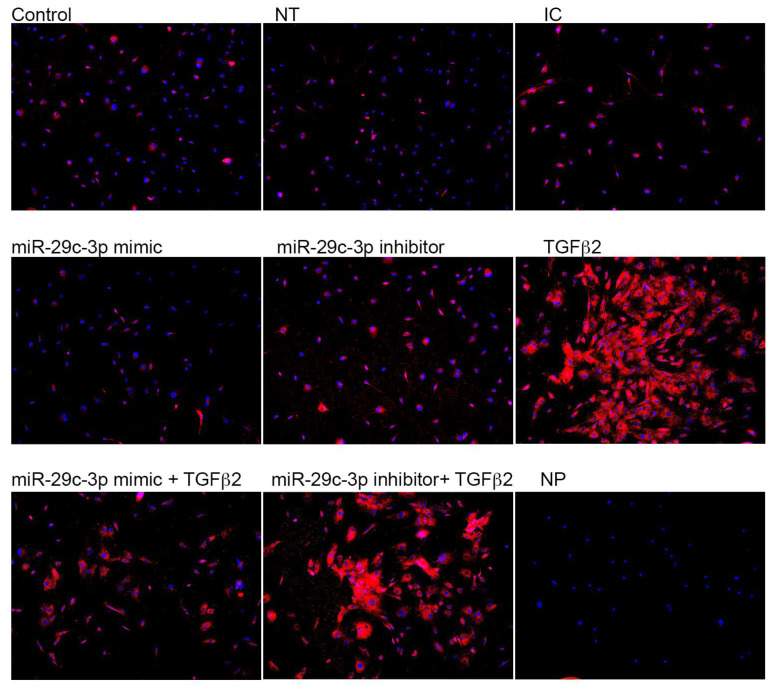
TGFβ2 and miR-29c-3p regulate collagen IV protein expression in LC cells. Primary human LC cells were transfected with miR-29c-3p mimic (10 nM), inhibitor (10 nM, inhibitor control (IC; 10 nM), or non-targeting (NT; 10 nM) control in the presence or absence or TGFβ2 (5 ng/mL) for 72 h. Cells were fixed and immunolabelled with anti-collagen type IV antibody (red). Nuclei (blue) were stained with DAPI. NP = no primary Ab control. Representative images are shown. Images taken at 100× magnification.

We next sought to validate the effects of TGFβ2 and miR-29c-3p on the predicted targets of miR-29c. Previously, we showed that collagen expression was increased with TGFβ2 in LC cells compared to control [18]. Therefore, we analyzed the potential effects of using a miR-29c-3p mimic and inhibitor on *TGFβ2*-induced collagen types I and IV expression by immunocytochemistry (Figure 5 and Figure 6). *TGFβ2* induced the expression of collagens type I (Figure 5) and IV (Figure 6) in LC cells. Transfection with miR-29c mimic prevented the *TGFβ2*-induced expression of collagen types I and IV. In contrast, the inhibition of miR-29c did not block the upregulated *TGFβ2*-induced collagen I and IV protein expression. This suggests that miR-29 may regulate *TGFβ2* signaling and synthesis of ECM proteins. Obtaining additional LC cells from glaucoma patients for the immunostaining was a limiting factor in our experiments.

## 3. Discussion

We report the first analyses of miRNA expression in human LC cells. We have shown that miR-29c-3p is downregulated in glaucomatous and TGFβ2 treated LC cells. In addition, using miRNet, a web-based tool, to investigate the potential function of miR-29c-3p, we show that miR-29c-3p targets several ECM proteins and is heavily involved in tissue remodeling. In vitro analysis shows that inhibition of miR-29c-3p promotes collagen types I and IV synthesis, while overexpression of miR-29c-3p led to a decrease in protein expression. Our findings suggest that downregulation of miR-29c-3p in glaucoma and TGFβ2 treated LC cells disrupts the ONH ECM and may affect the laminar tissue homeostasis, leading to pathogenic damage to the glaucomatous ONH.

Using TargetScan and miRNet, we were able to predict miRNA-target interactions for the miR-29 family. The anti-fibrotic miR-29 family targets the mRNA of several ECM and ECM-related proteins, including the collagens, bone morphogenetic protein 1(BMP1), and lysyl oxidase, suggesting that it plays a role in ECM and tissue homeostasis. Interestingly, TGFβ2 induces the expression of BMP1 and lysyl oxidase in trabecular meshwork cells [26,27]. BMP1 converts secreted ECM and ECM-related precursor proteins into mature functional proteins. Furthermore, miRNA-29b negatively regulated BMP1 and prevented the processing and synthesis of ECM genes in trabecular meshwork cells [22]. Also, lysyl oxidase is responsible for increased post-translational covalent cross-linking of ECM proteins. Moreover, Zhang et al. reported that TGFβ decreased miRNA-29b and induced lysyl oxidase expression in an immortalized human hepatic stellate cell line [28]. These data suggest that the miRNA-29 family targets BMP1 and lysyl oxidase and is involved in the tissue remodeling observed in glaucoma. The three main members of miR-29 (miR-29a, miR-29b, and miR-29c) have several common predicted targets; however, they may have tissue or cell-specific expression patterns and functions. ONH cells, including LC cells, are thought to contribute to glaucomatous tissue remodeling of the LC; therefore, it is necessary to explore the expression and role of fibrosis-related miRNAs. We found that all three members of the miR-29 family were downregulated in glaucomatous LC cells; however, only miR-29a-3p and miR-29c-3p were statistically significant. Similarly, these miRNAs were downregulated in TGFβ2 treated LC cells, and we also confirmed through qPCR analysis that miR29c-3p is downregulated in LC cells treated with TGFβ2. Overall, our results suggest that TGFβ2 may negatively affect the expression of miR-29 in LC cells, and that this miRNA therefore appears to be biologically relevant to ONH cells. Since miR-29 is negatively associated with ECM synthesis, inhibition of this miRNA may contribute to the glaucomatous tissue remodeling in the ONH. We need to better understand the expression pattern of miR-29, including the cellular localization and the functional roles of miR-29 in the normal and glaucomatous ONH.

Our results, along with those of other independent investigators, have shown that TGFβ2 remodels the ECM of the ONH and eventually leads to progressive damage to RGC axons. Our results show that TGFβ2 signaling and miR-29c-3p are important in regulating ECM synthesis, and that inhibition of miR-29c-3p may lead to aberrant ECM synthesis. Previous studies have shown that there is increased expression of TGFβ2 in the glaucomatous ONH and that elevated TGFβ2 is associated with increased ECM deposition [18,19,29]. We show that in the absence of TGFβ2 signaling, overexpression of miR-29c-3p leads to inhibition of collagen type IV synthesis in cultured LC cells. TGFβ2 treatment increases collagen types I and type IV expression in LC cells. However, the miR-29c-3p mimic antagonizes this effect by decreasing protein expression of collagen types I and IV. We also used an miR-29c-3p inhibitor in both the presence and absence of TGFβ2, and observed increased expression of collagen types I and IV compared to controls. We observed intracellular collagen in LC cells due to the short miRNA transfection protocol, as extracellular collagen expression takes time to get synthesized, secreted, and assembled.

These data collectively suggest that miR-29c-3p is an important regulator of ECM synthesis and may regulate TGFβ2 signaling in LC cells. Future studies will determine how miR-29c-3p regulates TGFβ2 signaling, either through inhibition of TGFβ ligands, TGFβ receptors, receptor SMADs, or by directly inhibiting the synthesis of ECM proteins [30].

Further studies may determine how TGFβ2 regulates miRNA expression in LC cells. For example, evaluating whether TGFβ2 signaling directly inhibits transcription of miR-29c or affects the processing of miR-29c [31]. It is possible that TGFβ2 signaling leads to the recruitment of transcription factors to the miRNA promoter, which can affect the transcription of miRNAs [24]. Alternatively, TGFβ2 signaling may affect the miRNA processing complex to alter primary, precursor, and mature miRNAs. Designing primers to the hairpin loop, precursor and mature miRNAs would be a way to quantify any differences [32,33]. To address what occurs first, miRNA or TGFβ2 dysregulation, we need to look at the response to earlier events in glaucomatous optic neuropathy such as elevated intraocular pressure (IOP). Models predict IOP-related stretch and compression of the laminar neural tissue, LC, sclera, and pia mater [34]. As IOP levels increased, the cells within the ONH reached peak strain (15%) at 50mmHg [34]. Further studies have been performed to investigate gene and protein expression changes in LC cells and astrocytes exposed to mechanical stretch. LC cells exposed to 15% stretch using the Flexcell system resulted in upregulation of ECM gene expression, including elastin, collagens, lysyl oxidase, and TGFβ2 [15]. Other studies analyzed the effects of increasing stretch by 0%, 3%, and 12%, on ONH astrocytes and LC cells [20,35]. In LC cells, protein synthesis increased from 3% to 12% stretch, suggesting LC cells are mechanosensitive and respond to mechanical strain [20]. To determine whether miRNAs are dysregulated in response to mechanical strain, we could analyze miRNA expression in response to stretch at low and high strain conditions. It may be possible that stretch dysregulates miRNAs and leads to enhanced TGFβ signaling.

## 4. Materials and Methods

### 4.1. Cell Culture and TGFβ2 Treatment

LC cell strains were derived from normal and glaucomatous human donor eyes obtained from the Lion Eye Institute for Transplant and Research (Tampa, FL) as previously described [15]. Acquisition of donor eyes closely followed the tenets of Helsinki with donor and/or family written consent. Donor eyes were anonymized to prevent donor identification. Primary human glaucoma (*n* = 4) and non-glaucomatous (*n* = 3) LC cell strains were generated from human lamina cribrosa explants and characterized and maintained as previously described [15]. Briefly, lamina cribrosa cells were characterized by negative expression for GFAP and positive expression for α-SMA and laminin [15]. LC cells were maintained in Ham’s F10 medium (Sigma Aldrich, St. Louis, MO, USA) supplemented with 10% fetal bovine serum (Atlas Biologicals, Fort Collins, CO, USA), glutamine (0.292 mg/mL), and penicillin (100 units/mL) and streptomycin (0.1 mg/mL) (Thermofisher Scientific, Waltham, MA, USA). The medium was changed every 2–3 days. Cell cultures were maintained at 37 °C with 5% CO_2_ within a humidified incubator. The ages of the normal donors were 56 years, 70 years, 74 years, and 82 years, while the ages of the glaucoma donors were 66 years, 73 years, and 97 years.

### 4.2. miRNA PCR Array

To evaluate glaucoma associated changes in LC cell miRNA expression, we seeded non-glaucomatous and POAG LC cells into wells of a 6-well plate in medium containing serum. To determine changes in miRNA expression due to TGFβ2 treatment, we also seeded non-glaucomatous LC cells into wells of a 6-well plate in medium containing serum, and when the cells reached 100% confluency, we replaced the medium with serum-free medium for 24 h. The next day, the cells were treated with recombinant TGFβ2 (5 ng/mL) (R&D Systems, Minneapolis, MN, USA) or vehicle control in serum-free medium. According to the manufacturer’s guidelines, total RNA was isolated using QIAzol and the miRNeasy mini kit (Qiagen, Germantown, MD, USA). RNA was quantified using the Nanodrop 2000 (Thermofisher Scientific, Waltham, MA, USA), and the purity/quality of RNA was assessed by 260/280 and 260/230 ratios. miRNA cDNA was synthesized using the HiSpec buffer and miScript II RT kit (Qiagen, Germantown, MD, USA). The thermoprofile parameters used were 37 °C for 60 min and 95 °C for 5 min. cDNA was diluted to 200 μL and we performed mature miRNA profiling using the miScript miRNA PCR array for Human Fibrosis (MIHS-117Z, Qiagen, Germantown, MD, USA) that includes 88 mature human miRNA primers. PCR was performed on the CFX96 real-time PCR system (Bio-Rad Laboratories Hercules, CA, USA). The thermoprofile parameters were 95 °C for 30 s, followed by 40 cycles of 95 °C for 10 s, 60 °C for 30 s, and concluded with a melting curve step. Representative data are shown on volcano plots. Statistical analysis was performed using Qiagen’s data analysis software (https://dataanalysis.qiagen.com/mirna/arrayanalysis.php) (accessed on 24 June 2020). The miRNAs included in this array target the mRNA of profibrotic, anti-fibrotic, signal transduction, epithelial-mesenchymal transition and ECM genes. For the full miRNA list, visit Qiagen’s website (https://geneglobe.qiagen.com/product-groups/miscript-mirna-pcr-arrays) (accessed on 24 June 2020).

### 4.3. In Vitro Transfection of LC Cells Using miRNA Mimics and Inhibitors

LC cells were plated 24 h before transfection in Ham’s F10 medium and transfected at a density of ~50–60% confluency (HiPerFect; Qiagen, Germantown, MD, USA) following the manufacturer’s instructions. In brief, 10 nM of miRNA-29c-3p mimic or miRNA-29c-3p inhibitor with 2.5uL HiPerFect were diluted in 1mL serum-free OPTIMEM medium(Invitrogen), then incubated for 15 min at room temperature and transferred to the appropriate well with LC cells. The cells were then incubated overnight at 37 °C in 5% CO_2–_95% air. The efficiency of the transfection was confirmed by qPCR. Negative controls consisted of cells with no treatment, non-targeting control for mimic experiments, and inhibitor control (Qiagen, Germantown, MD, USA) for inhibitor experiments. The miRNA-29c-3p mimic are double-stranded RNA oligonucleotides designed to mimic endogenously mature miRNA-29c activity. The miR-29c-3p inhibitors are single-strand RNA oligonucleotides designed to inhibit miRNA activity.

### 4.4. RNA Isolation and qPCR

For miRNA analysis, total RNA was isolated using a miRNeasy isolation kit (miRNeasy mini kit, Qiagen, Germantown, MD, USA) according to the manufacturer’s guidelines. Using 200 ng RNA, miRNA cDNA was synthesized by reverse transcription (HiSpec buffer and miScript II RT kit, Qiagen, Germantown, MD, USA). The thermoprofile parameters used were 37 °C for 60 min and 95 °C for 5 min. cDNA was diluted to 200uL to perform qPCR reactions in a 25 μL mixture containing miScript SYBR Green, miScript primer, miScript universal primer, RNase free water and cDNA. The miR-29a-3p (5′UAGCACCAUCUGAAAUCGGUUA) and miR-29c-3p (5′UAGCACCAUUUGAAAUCGGUUA) primer sequences were obtained from Qiagen. The expression of miRNA was normalized to SNORD 95 using the ΔΔ cycle threshold (CT) method.

For mRNA analysis, total RNA was isolated using the RNeasy kit (Qiagen, Germantown, MD, USA). RNA was quantified using the Nanodrop 2000 (Thermofisher Scientific, Waltham, MA, USA) and purity/quality of RNA assessed by 260/280 and 260/230 ratios. cDNA was synthesized from total RNA (500ng) by reverse transcription using the iScript supermix (Bio-Rad Laboratories) according to the manufacturer’s instructions. Q-PCR reactions were performed in a 20μL mixture containing 1μL of the cDNA with 1X SYBR Green Supermix (Bio-Rad Laboratories), using the following parameters: 95 °C for 5 followed by 40 cycles of 95 °C for 15 s, and 72 °C for 15 s (CFX96 System and CFX Manager Software; Bio-Rad Laboratories). Primers were designed using Primer3 software (Whitehead Institute, Massachusetts Institute of Technology, Cambridge, MA) for collagens 1a1 and 4a1. The expression of mRNAs was normalized to GAPDH using the ΔΔ CT method. Error bars show the SD. A student’s *t*-test, *n* = 3 technical replicates, * *p* < 0.05.

*Collagen 1a1* Forward 5′-AGCCAGCAGATCGAGAACAT-3′

*Collagen 1a1* Reserve 5′-TCTTGTCCTTGGGGTTCTTG-3′

*Collagen 4a1* Forward 5′-ATAGACGGATATCGGGGGCCT-3′

*Collagen 4a1* Reverse 5′- GGATTTGAAAAAGCAATGGCACTC-3′

*GAPDH* Forward: 5′-GGTGAAGGTCGGAGTCAAC-3′

*GAPDH* Reverse: 5′-CCATGGGTGGAATCATATTG-3′

### 4.5. Immunocytochemistry

LC cells were plated at a density of 8000 cells/well in a 24 well plate with glass coverslips and incubated in Ham’s F10 medium at 37 °C and 95% air. The following day, the cells were transfected with 10nM of miRNA mimics, inhibitors, or controls in OptiMem. The next day, the cells were treated with or without TGFβ2 for 48 h in Ham’s F10 medium. Cells were fixed in 4% paraformaldehyde in Dulbecco’s phosphate-buffered saline (PBS; Sigma-Aldrich, St Louis, MO, USA) for 10 min at room temperature. Cells were permeabilized with 0.2% Triton-X 100 at room temperature for 20 min and then blocked with 10% donkey serum in PBS superblock for 1 h at room temperature, followed by primary antibody (Table 1) (diluted 1:100 in Superblock PBS) incubation overnight at 4 °C under dark conditions. The next day, the cells were washed three times with PBS for 5 min each, followed by incubation with the appropriate secondary antibody conjugated to a fluorescent 594 or 488 dye (Invitrogen; diluted 1:200 in Superblock PBS) for 1 h at room temperature in dark conditions. Cells were then washed three times with PBS for 5 min each, followed by two quick rinses with dH_2_O. Coverslips were mounted carefully with DAPI prolonged gold (Invitrogen) and left to dry at room temperature for 24 h in dark conditions. Controls consisted of the omission of primary antibodies. Images were captured using the Nikon Eclipse TieU microscope (Melville, NY, USA) containing the Nuance FX imaging system (CRI, Burlington, MA, USA).

### 4.6. Statistical Analysis

A student’s *t*-test was performed to determine statistical significance between two groups. A one-way ANOVA and Tukey’s multiple comparison test was performed to determine statistically significant differences between three or more independent groups. GraphPad Prism 9.0 (GraphPad Software, San Diego, CA, USA) was used for data analysis. A value of *p* < 0.05 was considered to be significantly different.

## 5. Conclusions

In summary, we have used an in vitro cell culture to determine the expression of miRNAs in glaucomatous, non-glaucomatous, and TGFβ2 treated human LC cells. Our data showed downregulation of the miRNA-29 family in glaucomatous LC cells compared to normal healthy LC cells. Moreover, there was a decrease in the expression of the miRNA-29 family upon exogenous treatment with TGFβ2. We believe there is an interaction between the miR-29 family and mRNAs encoding the collagen genes in LC cells. Moreover, it is predicted that the miR-29 family target multiple collagen genes using the TargetScan database and miRNet network analysis. Lastly, transfecting LC cells with miR-29c-3p inhibited TGβ2 induced collagen I and IV. The dysregulation of TGFβ2 signaling and aberrant miR-29 expression may contribute to LC tissue remodeling in glaucoma. TGFβ2 can change miR-29c-3p expression, and may eventually contribute to increased ECM deposition in the glaucomatous LC. Our data suggest that restoration of miR-29c expression in the ONH may restore homeostatic TGFβ2 signaling and ECM turnover.

## Figures and Tables

**Table 1:** Antibody list used for immunocytochemistry staining.

Antibody	Source	Dilution
Rabbit anti-Collagen I	Abcam	1:200
Rabbit anti-Collagen IV	Abcam	1:200
Donkey anti-Rabbit 488	Invitrogen	1:500
Donkey anti-Rabbit 594	Invitrogen	1:500

## Data Availability

All expression data are provided in Supplementary Tables. Data also have been deposited in GEO.

## Supplementary Materials

The following are available online at https://www.mdpi.com/article/10.3390/ijms22126178/s1.
